# Case of Deafness Probably Caused by Amalgam Fillings

**Published:** 1883-03

**Authors:** H. Purfield


### Case of Deafness Probably Caused by Amalgam Fillings.

BY H. PURFIELD.

In February or March, 1880, I had three cavities which were filled with amalgam; two large cavities in the second and third upper molars, left side, and one, smaller, in second lower molar, same side. Had for many years been troubled with naso-pharyngeal catarrh—result of measles, causing a slight dullness of hearing. After the insertion of the amalgam fillings, this dullness, previously but little noticed, rapidly increased to deafness in the *left* ear, so that within a few months the ticking of a watch could with difficulty be distinguished, even when placed close to the orifice of the ear.

A medical examination about that time showed that the eustachian tube had become almost closed, and that the secretion of cerumen had stopped. Treated for catarrh, with some improvement in that respect, but none in hearing.

The right ear, which had remained in about a normal condition up to this time, now became gradually affected until a watch could not be heard to tick beyond a few inches.

The following winter, owing to trouble with one of the teeth that had been filled—the second molar, upper,—the filling was removed, and treatment commenced for disease of the antrum, which resulted in a complete cure of that affection in a short time.

After this filling—which, as stated before, was a large one—had been removed, the progress of deafness in the right ear was sensibly retarded, though not entirely stayed. In addition to deafness, neuralgic symptoms had appeared, affecting the supra-orbital and other nerves; particularly felt upon unusual mental or long protracted physical effort. In November, 1882, about two years and eight months after insertion of amalgam fillings, their removal was advised, which was done, and the cavities—two of them—were filled with gold. Immediately upon the removal of the amalgam a sense of relief was experienced, which, in spite of the sensitiveness of the newly filled teeth, has continued and increased to the present time (January, 1883). Mental exercise is now not attended with the difficulty experienced before, and the throbbing and drumming in the ears, upon a straining effort to hear, have greatly diminished. The improvement in hearing, though quite perceptible in the left ear (the one most affected) is not yet as marked as is the lessening of the nervous irritability. There is also noticed a decreased susceptibility of catarrhal condition of the parts affected.

